# Neural basis of approach and avoidance responses to food in 12-month-old infants following emotional state changes

**DOI:** 10.1016/j.dcn.2025.101656

**Published:** 2025-12-07

**Authors:** Liam R. Chawner, Sayaka Kidby, Arkadij Lobov, Alejandra Sel, Maria L. Filippetti

**Affiliations:** Centre for Brain Science, Department of Psychology, University of Essex, Colchester CO4 3SQ, UK

**Keywords:** Emotional eating, Infants, Feeding practices, Interoception, Emotion regulation, Approach/avoidance

## Abstract

Emotional Eating (EE) behaviours may emerge throughout childhood as a function of maladaptive interoceptive abilities, where eating occurs in response to emotional states rather than to satisfy hunger signals. Genetic and neurobiological factors contribute to EE, indicating that underlying neural mechanisms may precede the manifestation of these behaviours. We examined the neural processes associated with the early development of EE. Twelve-month-old infants attended the lab and ate lunch until satiation before being exposed to a frustration-inducing task. While wearing an EEG cap, infants viewed pictures of liked foods and non-foods. We measured infants’ behavioural reactivity to the frustration task, Frontal Alpha Asymmetry (FAA) indicating approach-avoidance responses to food and non-food stimuli, and collected parent-reported data on infant appetitive traits and temperament, and feeding practices. At low levels of emotional reactivity to frustration, infants showed more approach to non-food stimuli, whereas for some infants with higher emotional reactivity, stronger FAA approach activity was observed towards food stimuli. Additionally, parental use of feeding to regulate emotions predicted higher FAA approach responses to both food and non-food stimuli. These results suggest that infants’ neural responses to a change in emotional state are associated with approach-avoidance tendencies towards food and non-food stimuli, before EE behaviours emerge. However, associations between food approach tendencies and parental influences at 12months remain unclear.

## 1 Introduction

During infancy and toddlerhood, children begin to associate changes in their bodily sensations with external objects and events. For example, they learn to attend to stimuli that elicit positive internal sensations, such as comfort or pleasure, while avoiding those that cause aversive bodily states, such as discomfort or distress. This process helps to maintain physiological balance and is guided by interoception; the ability to sense, integrate, and interpret information about the state of the inner body (Berntson and Khalsa, 2021, Khalsa et al., 2018).

Interoceptive processing not only allows the individual to monitor internal bodily states but also shapes how external stimuli are evaluated in relation to current needs. For example, the value of a milk stimulus (e.g. the breast or a bottle) to an infant becomes greater during hunger compared to when satiated. In turn, pleasant internal sensations arising from feeding would increase the likelihood of the infant to seek (approach) the bottle or breast in the future.

At the neural level, the insular cortex, which plays a central role in integrating interoceptive information, is thought to influence approach and avoidance behaviours by translating bodily states into reward or loss outcomes (Paulus and Stewart, 2014). Through these mechanisms, interoception provides a bridge between internal physiological regulation and value-based decision-making and reward-related behaviour (Cabanac, 1971, Paulus, 2007). Indeed neuroimaging studies in adults have consistently demonstrated that hunger modulates food-related activity in the insula, as well as in brain regions implicated in reward and motivation (e.g. Goldstone et al., 2009, LaBar et al., 2001, Uher et al., 2006).

Given the value and rewarding nature of stimuli, maladaptive associations with internal sensations have potential to develop. One such maladaptive association is Emotional Eating (EE), which occurs when individuals learn to associate emotional states – rather than physiological hunger – with food intake (Bongers et al., 2015). EE exemplifies how disruptions in the interplay between interoception and reward systems result in maladaptive food-related behaviours. When a child learns to associate food with successful emotional regulation, the value of a food stimulus will increase under emotional distress and lead to approach behaviours towards food even in the absence of hunger. Indeed in adults, the likelihood to approach rewarding stimuli has been positively associated with EE (for a review see Sutton et al., 2022).

Early dyadic interactions can compound this effect. Research studies on the development of feeding and eating behaviours have shown that both parental and child factors can either mitigate or exacerbate susceptibility to EE. For example, parental use of food as a reward and restrictive feeding practices at 3–5 years of age have been found to predict higher EE at 5–7 years (Farrow et al., 2015). Additionally, children whose parents offer snacks when they are bored are more likely to EE (Stone et al., 2023). Importantly, this association is also predicted by the child’s own temperament, suggesting complex and bidirectional relationships. Studies have shown that parental use of coercive control practices is associated with poor emotion regulation abilities and increased EE in 4–5-year-olds (Baker and Fuglestad, 2024). Altogether this research suggests that both parental and child factors may interact to influence the development of EE.

While the vast majority of the research on the development of EE has focused on behavioural evidence in school-aged children (although see Rodgers et al., 2014; Temmen et al., 2021), little is known about its developmental origins (Chawner and Filippetti, 2024). Examining the neural mechanisms underlying EE may reveal key predictors that contribute to susceptibility to this behaviour. This is especially relevant given the evidence suggesting that genetic effects on EE are small at 1–5 years, but increase at 12 years old, when the child has more control over their food decisions (Madhavan et al., 2025).

At the neural level, asymmetrical activity in the frontal cortex (FAA), quantified by alpha power using EEG, is associated with adults’ approach and avoidance motivation in food-related contexts (Kirsten et al., 2022, Ochner et al., 2009, Winter et al., 2016). Generally, while relatively stronger activity of the left over the right frontal hemisphere indicates the tendency to approach a stimulus, relatively stronger right frontal activity is associated with an increased avoidance or avoidance motivation (Coan and Allen, 2004, Harmon-Jones et al., 2010). FAA has also been widely used in infancy research as an index of emotion tendencies and temperament (Fox, 1994), showing that higher right (relative to left) FAA is associated with negative emotions, poorer emotion regulation skills, and avoidance behaviours, whereas higher left (relative to right) FAA is indicative of positive emotions, better emotion regulation abilities, and approach behaviours (for a review see Cuevas and Bell, 2022).

Individual differences in children’s behavioural regulatory abilities have been implicated to EE and food approach tendencies (Liew et al., 2020), however these associations are only apparent once such behaviours arise. Neural markers can allow us to identify whether infants are drawn towards different types of food in response to a change in emotional state, without the need for an overt behavioural response. In this study, we sought to examine how parental feeding practices and infant temperament may influence 12-month-old infants' approach towards, or avoidance from, food in response to a perturbation of internal (emotional) state (a frustration task) and in the absence of hunger. Most of the research on FAA has focused on frontal EEG asymmetry as a trait of emotional and motivational tendencies (e.g. Harmon-Jones and Gable, 2018), however task-related FAA has been also used to study state-related activity reflecting the interplay between a person's regulatory abilities and the specific emotional demands of the environment (Coan and Allen, 2004, Diaz and Bell, 2012, Kirsten et al., 2022). We reasoned that FAA resulting from an interaction between the infant’s regulatory abilities and the situational context may index a predisposition to EE. Therefore, in the current study we collected both dispositional (at rest) and task-related FAA. We hypothesised that 1) infants who displayed higher behavioural reactivity in response to a mild stressor would show higher left (relative to right) FAA in response to food vs. non-food stimuli, suggesting a stronger motivational approach to food stimuli in response to mild stress; 2) infants whose parents reported higher food responsiveness would more prominently show left frontal alpha asymmetry to foods than to non-foods; and 3) infants whose parents more frequently practice emotional feeding and/or reward feeding would more prominently show left frontal alpha asymmetry to foods than to non-foods.

## 2 Methods

### 2.1 Participants

Ninety-three 12-month-old infants (51 females, M_age_ = 374 days, *SD*_*age*_ = 14.5 days) were recruited to participate in this study. The infants were recruited via phone calls and emails from a database consisting of parents from the East of England, UK, who had previously expressed an interest in taking part in psychological research. Full demographic information is presented in Table 1. All infants invited to participate were born full term (37–41 weeks).

**Table 1:** Participant demographic information.

**Participant characteristics**	
Total infants, Female (%)	93, 51 (55)
Ethnicity of infant, N (%)	
White	85 (91.4)
Other	8 (8.6)
Household income, N (%)	
Up to £24,999	5 (5.4)
£ 25,000 to £ 49,999	22 (23.7)
£ 50,000 to £ 74,999	29 (31.2)
£ 75,000 to £ 99,999	15 (16.1)
£ 100,000 or more	17 (18.3)
Prefer not to answer	5 (5.4)
Parental education, N (%)	
Secondary school	3 (3.2)
Further education (A-levels, BTEC, etc.)	20 (21.5)
Undergraduate degree / vocational qualification	40 (43.0)
Postgraduate degree	23 (24.7)
Other	7 (7.5)
Infant zBMI, Mean (SD)	0.69 (1.5)

An a-priori power analysis (using the R package ‘pwr’) indicated that 85 participants were required for a mediation analysis to detect a medium effect size (f^2^ =.18) with a significance level of.05 and power of.80. Although 93 participants attended the lab sessions, this minimum number was not met in the final sample for the following reasons: 1) EEG data had to be excluded due to signal quality problems (e.g. due to the infant pulling on the EEG net, excessive movement, or poor contact with the scalp, N = 10), 2) because the infant refused to wear the EEG net (N = 9), 3) because infants displayed excessive fussiness or crying during the EEG task (N = 22), and 4) because of technical difficulties (N = 3). Furthermore, 7 infants did not have full Lab-TAB data due to becoming fussy/overly frustrated during this task. Overall, 41 infants had usable FAA food task data (with no other missing data, e.g. Lab-TAB, lunch intake) and 43 infants had useable resting state FAA data (with no other missing data). Of these infants, 37 had usable data for both food task and resting state FAA. These samples are used in the final analyses where all available complete data have been utilised.

Participants received a children’s storybook for taking part. Prior to recruitment, ethical approval for the study was obtained from the University of Essex Research Ethics Committee (ETH2122-0126). Written informed consent was obtained from the infant's caregiver prior to the start of the experiment. The study preregistration and data can be viewed on OSF: osf.io/jby4g.

### 2.2 Measures

*Online survey.*

Parents completed an online survey containing demographic questions, along with questionnaires addressing the child’s eating traits (Child Eating Behaviour Questionnaire: CEBQ, Wardle et al., 2001), parental feeding practices (Comprehensive Feeding Practices Questionnaire: CFPQ, Musher-Eizenman and Holub, 2007) and the child’s temperament (Infant Behaviour Questionnaire, Very Short form: IBQ-R, Putnam et al., 2014). From the CEBQ, subscales included in analyses were satiety responsiveness (α = 0.57, AVE = 0.30), food responsiveness (α = 0.65, AVE = 0.37) and emotional overeating (α = 0.56, AVE = 0.58). Subscales included from the CFPQ were use of food to regulate emotions (α = 0.63, AVE = 0.51) and use of food as a reward (α = 0.63, AVE = 0.68). Lastly, the IBQ-R subscales included surgency (α = 0.70, AVE = 0.24), negative affect (α = 0.81, AVE = 0.35) and effortful control (α = 0.75, AVE = 0.29). Parents were also asked to rank a selection of high energy density (HED) and low energy density (LED) foods in order of their child’s preference (see *Food Task* below).

*Lunch.*

Parents provided their child’s lunch because, at the time of ethical approval and the study's commencement, COVID-19 restrictions prevented researchers from supplying food. Additionally, young children may be less likely to eat a standardised lunch, particularly if they are unfamiliar with the food items or the setting. Parents were also allowed to bring their own lunch if they usually ate with their child. All infants ate lunch in the lab. Lunch items were weighed and pictured before and after eating for subsequent estimation of calorie consumption. If food packaging was available, the nutrition information was used to estimate calorie consumption. If there was no packaging, energy was estimated using the “Composition of Foods Integrated Dataset” (McCance and Widdowson, 2014). Parents were instructed to feed their child as they usually would at home and not to worry about the time that it took their child to eat. This was to ensure that the child ate what they wanted and that slowness in eating did not impact them in becoming satiated. The parent signalled to the experimenter when they thought their child had finished eating and were full.

*The “attractive toy behind a barrier” Lab-TAB task*

The Lab-TAB “attractive toy behind a barrier” task (Goldsmith and Rothbart, 1996) was used to elicit an emotional perturbation (e.g. emotions of anger, sadness, or frustration) by the infant. The task began when the experimenter brought the attractive toy into the infant’s view, demonstrated how it worked and gave it to the infant. The attractive toy had a round bottom part so that it could wobble or spin around, but not fall over. It also had a bell inside that made noise when it was moved. The infant was then allowed to play with the toy for 15 s before the experimenter placed a transparent Plexiglas barrier (40 cm high x 40 cm wide) in front of them (within their reach). The experimenter then gently took the toy back and placed it directly behind the barrier. The toy was left behind the barrier for 30 s, after which the experimenter returned it to the infant. This procedure was repeated two more times for a total of three trials. In contrast with the Lab-TAB manual, the toy was *not* returned after the third trial, as the purpose of this episode was to elicit an emotional perturbation so that the influence of this perturbation on the infant’s subsequent EEG response to food stimuli could be assessed. The entire episode lasted for 2 min and 15 s.

*Food task*

**Fig. 1:**
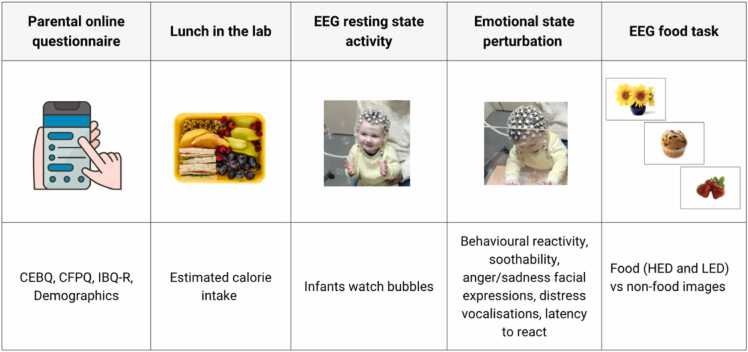
Experimental protocol of the study. Note. An overview of procedure tasks, order, and measures collected for the study. Before their lab visit, parents completed an online survey including demographics, and questionnaires addressing the infant’s eating traits, parental feeding practices, and child temperament. On the days of the lab visits, parents provided lunch for their infant and calorie intake was estimated for each participant. After lunch and a 5-min break, the participants underwent the EEG resting state activity, followed by the emotional state perturbation (the “Attractive toy behind the barrier” LAB-Tab task). Measures of behavioural reactivity, soothability, facial expressions, distress vocalizations, and latency to react were taken from each participant. This was immediately followed by the EEG food task, whereby participants watched images of food (high [HED] and low [LED] energy density) and non-food on a computer screen.

For the food task, experimental stimuli comprised of four picture categories: 6 liked HED food pictures, 6 liked LED food pictures, 6 animal pictures, and 6 plant pictures. Therefore, a total of 12 liked food pictures and 12 non-food pictures were selected for each infant. Within each food category, each infant watched three types of food with 2 different pictures for each food. Importantly, food pictures were individually pre-selected and tailored to the preferences of each infant. Parents ranked the three most liked LED and HED foods for each infant, ensuring hedonic appeal of the food stimuli. Pictures were taken from the food-pics database (Blechert et al., 2014). Examples of the pictures are displayed in Fig. 1**.** Image properties and statistics of the images used for the EEG food task are presented in Table 1S (Supplementary Material A).

The computer monitor used to display the experimental stimuli was 23” in size and located ∼90 cm from the participant's eyes. Throughout the whole experiment the infant’s attention to the display screen was monitored via a video camera located under the screen. The session began with a colour cartoon image presented in the middle of the screen for a random duration set between 1400 and 1800ms, which we used to attract the infant’s attention to the screen. Next, the food (HED and LED) and non-food (animal and plants) stimuli were each presented for 1000 ms in a pseudo-random order and for a total of 300 trials (75 trials for each stimulus condition). In between each trial, a colour cartoon image was displayed for a random duration between 1050 and 1450ms. The computer played the stimuli through Matlab (Mathworks), and a Macintosh computer recorded the EEG signal.

*EEG recording and processing*

Brain electrical activity was recorded via Hydrocel Geodesic Sensor Net (Electrical Geodesic Inc.), consisting of 128 electrodes evenly distributed across the scalp and referenced to the vertex. EEG was amplified with a 0.1–100 Hz band-pass filter and digitized at 500 Hz. The data was pre-processed off-line using NetStation 5.4.2 analysis software (Electrical Geodesic Inc.) and WTools (Ferrari et al., 2025). For both resting state and food task, continuous EEG data was low-pass filtered at 30 Hz using digital elliptical filtering. For both food task and resting state data, segments with eye movements and blinks were detected visually and rejected from further analysis. We manually excluded trials in which the infants were not attending and/or the caregivers were influencing the infants (e.g. by talking). For the resting state data, each participant had to have at least 30 2-second-long clean trials for the data to be included for further analyses (Vincent et al., 2021). Infants contributed to a mean of 48.77 artifact-free trials. For the food task, a minimum number of 5 trials per condition was required to carry out the baseline-corrected averaging analysis. This minimum number is based on similar previous infant research (de Klerk et al., 2015, Quadrelli et al., 2021). Infants contributed to a mean of 16.5 artifact-free trials for the food pictures and a mean of 16.12 artifact-free trials for the non-food picture. A paired-sample *t*-test showed that the number of trials did not significantly differ between conditions, *p* = 0.369.

Complex Morlet wavelets were computed at 1 Hz intervals in the 5–25 Hz range. The food task EEG data was segmented into 2050 ms trials, consisting of a 500 ms pre-stimulus baseline, a 950 ms analysis period, and 300 ms buffer on either side of the segment to eliminate distortion created by the wavelet transform (de Klerk et al., 2015). Infant alpha range has been identified within the 6–9 Hz frequencies (Marshall et al., 2002) and therefore we used this range to analyse FAA in this study. Activity in the 6–9 Hz-frequency range during the 500 ms baseline period was subtracted from the 950 ms analysis period. The continuous EEG resting state data was segmented into 1800 ms trials, consisting of a 1000 ms analysis period and 400 ms buffer on either side of the segment. No baseline correction was performed on the EEG resting state data.

### 2.3 Procedure

Before visiting the lab, parents completed an online survey. On arrival at the lab, infants had opportunity to play with toys and familiarise themselves with the environment whilst the experimenter explained the procedure to the parent. The parent then indicated when their infant was hungry or ready to eat lunch and both were taken to a room equipped with a high-chair and tray. The experimenter weighed and took pictures of all foods before and after eating. After lunch, the parent and their infant returned to the reception room for a 5-minute break. Next, participants were taken to the testing room by the experimenter. The parent was instructed to sit on a chair in front of a table (63 cm wide x 56 cm deep) with their infant sitting on their lap and facing the table. After placing the EEG on the infant’s head, the study began.

First, the resting state data acquisition took place. This part of the study lasted until the participant became fussy, or until 3 min of data was recorded. To keep the infants as still as possible, the experimenter played a bubble machine, modelling the procedure used in previous studies (e.g. (Anderson and Perone, 2018; Hane et al., 2008; Hane and Fox, 2006; Marshall et al., 2002)). The parent was asked not to talk nor interact with their baby during the experiment.

Following the resting state data acquisition, the experimenter conducted the Lab-TAB “attractive toy behind a barrier” task (Goldsmith and Rothbart, 1996). Lastly, caregivers were asked to move in front of the computer screen with their infant facing the monitor where they watched pictures of food and non-food items until they became fussy or bored, as judged by the experimenter. Infants were encouraged to watch the stimuli displayed on the monitor. Parents were asked to refrain from talking and interacting with the infant during the stimuli presentation unless the infant became fussy. If the parent talked to redirect the infant's attention to the screen or in case of fussiness or distraction, we excluded this chunk of data from the recording. The testing session lasted between 5 and 10 min, depending on the infant’s willingness to watch the stimuli. The whole visit took approximately 1 h and 30 min. At the end of the study, parents were fully debriefed of the study’s aims and the infant’s height and weight measured. See Fig. 1 for an overview of the study procedure.

### 2.4 Data analysis

*Behavioural data analysis*

Coding and scoring of the Lab-TAB “attractive toy behind a barrier” task was performed according to the Lab-TAB manual version 3.1 (Goldsmith and Rothbart, 1996). Infants’ behaviours were coded for intensity of facial expression of anger or sadness (0−3), distress vocalisations (0−5) and behavioural struggle (0−4) for both experimental and control conditions. It was also noted how long it took for the infant to react with a behavioural expression of anger or sadness (latency to react) and how long it took for the infant to stop their behavioural expressions (soothability). To score these behaviours, each Lab-TAB trial was split into six 5-second epochs. An average for each behaviour was then calculated for each of the three trials separately. The two trials with the highest reactivity scores (facial expression, vocalisation and struggle) were then used to create a composite variable of behavioural reactivity intensity of the infant. A higher composite score indicates higher emotional expression and reactivity. Three trained researchers who were blind to participants’ other variables (e.g., parental feeding style) coded the video recordings of all participants taking part in a larger research project from which the present data were also gathered (Chawner et al., 2025). Two researchers independently coded 26 videos. Inter-rater reliability for the same coding task, conducted by the same coders in a separate study, was determined using intraclass correlation (r = .94), indicating high agreement between observers.

*EEG data analysis*

For the resting state data, alpha asymmetry scores were calculated by subtracting the natural log of alpha power at the left hemisphere site (F3) from the natural log of alpha power at the right hemisphere site (F4) at the frontal scalp locations based on previous studies demonstrating that the alpha asymmetry between these two sites index a general response tendency of approach versus avoidance (Coan and Allen, 2004, Fox et al., 2001) and positive versus negative emotionality (e.g. Jones et al., 2009).

For the food task, given the lack of prior research using this approach, we had no prior assumptions about the timing of FAA effects, and therefore the identification of the time window for further analyses was guided by cluster-based permutation analysis (Supplementary Material B). The results of this analysis revealed increased alpha activity in the E24–E124 pair (i.e. increased FAA) for the contrast food vs non-food stimuli (Monte-Carlo p value = 0.046,) between 596 and 706ms (Supplementary Material Fig. 1S). Therefore, all subsequent analyses focused on this electrode pair and time window.

*Statistical analysis*

Statistical models were conducted using linear regression with cluster robust standard errors and confidence intervals (to account for repeated measures across conditions). Planned (preregistered) mediation analyses were not conducted due to insufficient power to adequately test these hypotheses. All statistical analyses were conducted using R version 4.2.3 using packages “tidyverse” v2.0.0, “lmtest” v0.9–40, “sandwich” v3.1–0 and “sjPlot” v2.8.15.

## 3 Results

### 3.1 Primary analyses

*Lunchtime food intake*

Infants consumed a variety of food items at lunch, resulting in varied energy intake (M = 170 kcal, SD = 110, Range = 25–532 kcal). Despite differences in meal consumption, parents reported that 67 % of infants ate about the same amount of food as they usually would eat at lunchtime, with 7 % of infants eating more than usual, and 26 % eating less than usual. Sensitivity analyses performed for the analyses below found no differences to outcomes when participants reported as eating less than usual were excluded from the analyses (Supplementary Material C; Table 2S).

**Table 2:** Predictors of FAA when presented with food and non-food stimuli.

	**Frontal Alpha Asymmetry (FAA)**				
*Predictors*	*Estimates*	*Std. Error*	*CI*	*t-value*	*p-value*
(Intercept)	0.45	0.16	0.14, 0.75	2.74	0.008
Estimated lunch calorie					
intake	0.00	0.00	0, 0	0.89	0.376
Satiety responsiveness (CEBQ)	-0.15	0.03	-0.21, −0.08	-4.29	< 0.001
Food responsiveness (CEBQ)	-0.12	0.04	-0.2, −0.04	-2.84	0.006
Emotional overeating (CEBQ)	-0.10	0.08	-0.25, 0.05	-1.30	0.196
Feeding to regulate emotions (CFPQ)	0.19	0.05	0.09, 0.29	3.69	< 0.001
Use of food as a reward (CFPQ)	0.08	0.05	-0.02, 0.17	1.55	0.126
Behavioural reactivity (Lab-TAB)	-0.03	0.01	-0.05, 0	-1.82	0.073
Condition (Food) [Ref: Non-Food]	-0.47	0.13	-0.71, −0.23	-3.69	0.006
Behavioural reactivity (Lab-TAB) × Condition (Food)	0.06	0.02	0.02, 0.11	2.86	0.006
Observations				N = 41	
R^2^ / R^2^ adjusted				0.304 / 0.217	
F			F(9,72) = 3.494, p = 0.001		

*Task-related FAA*

**Fig. 2:**
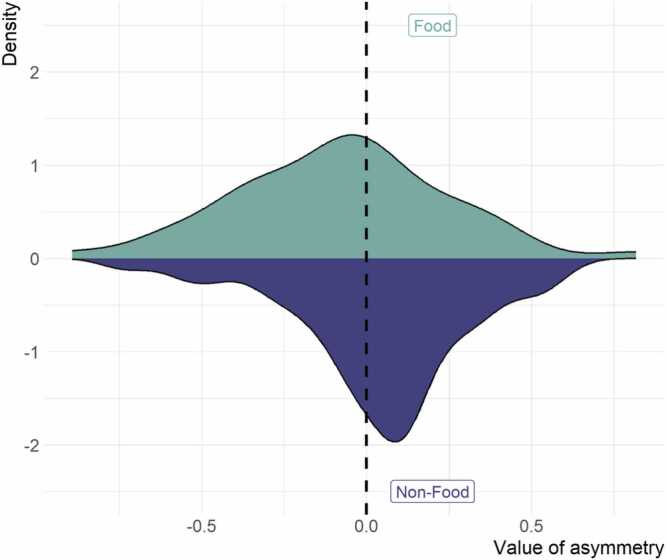
Density of FAA values for both food and non-food stimuli. Note. Density of FAA values for food (green) and non-food (blue) stimuli.

First, we tested whether averaged FAA responses differed between conditions. Task-related FAA responses were lower in the food (M = −0.08, SD = 0.32) compared to the non-food stimuli (M = 0.04, SD = 0.27) condition, *t*(48) = 2.05, *p* = 0.046 (Fig. 2).

Next, we examined predictors of FAA (Table 2). The model illustrates that when controlling for child appetitive traits and parental feeding practices, FAA for food stimuli (condition) showed stronger right-than left hemispheric cortical activity than FAA for non-food stimuli. Overall, this indicates lower approach activity towards food stimuli. Additionally, the CEBQ subscales of satiety responsiveness and food responsiveness, but not emotional overeating, were found to predict reduced approach tendencies. No interactions were found between these subscales and experimental condition.

**Fig. 3:**
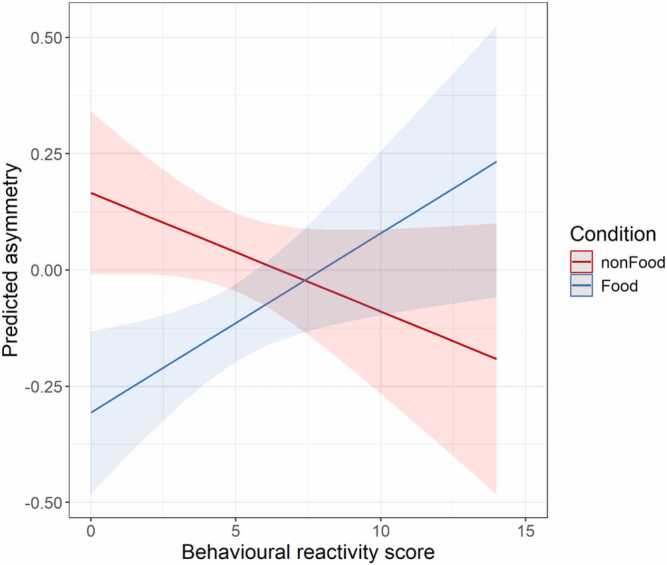
Interaction plot between Behavioural reactivity score and Condition on predicted FAA. Note. Plot depicting the significant interaction between behavioural reactivity score (x axis) and condition (y axis) on predicted FAA. Food FAA responses are displayed in blue, whereas non-food FAA responses are displayed in red.

Estimated lunch energy intake and CFPQ use of food as a reward were not associated with FAA. However, parental use of feeding to regulate emotions (CFPQ) was associated with stronger left-than right hemispheric cortical activity, suggesting that infants whose parents reported to use food to soothe their child displayed more approach tendencies across conditions in the EEG task. Lastly, the results show a significant interaction between behavioural reactivity (as assessed during the “attractive toy behind a barrier” Lab-TAB task) and the food stimuli condition (Fig. 3). This suggests that when behavioural reactivity is low, infants are more inclined to approach non-food stimuli (stronger left-than right hemispheric cortical activity), while infants who displayed higher behavioural reactivity had increased approach tendencies towards food stimuli compared to the non-food condition. However, larger variability in approach to food and non-food stimuli at higher levels of behavioural reactivity suggest that increased approach tendency to food stimuli may only be displayed by some infants.

### 3.2 Exploratory analyses

As an exploratory analysis, we tested the relationship between resting state (baseline) FAA activity and other temperament variables. Behavioural reactivity (Lab-TAB), surgency, negative affect, and effortful control (all measured using the IBQ-R) were not associated with resting FAA (Supplementary Material D; Table 3S). When tested alongside parental feeding practices and child eating traits, no variables were found to be predictive of resting state FAA activity (Supplementary Material D; Table 4S). Additionally, resting state FAA activity did not predict task-related FAA (β = −0.001, *p* = 0.99), and we did not find any interaction between resting state FAA and experimental condition (β = −0.098, *p* = 0.66) in predicting task-related FAA.

## 4 Discussion

In the current study, we examined how parental feeding practices and child temperament influence 12-month-old infant’s neural responses to food and non-food stimuli following an induced emotional perturbation. By using an EE framework, this study offered a unique lens through which to explore approach and avoidance tendencies associated with changes in physiological state and external stimuli. Existing research suggests that EE emerges in early childhood and increases with age (Ashcroft et al., 2008, Derks et al., 2019). Our results suggest that both infant temperament (higher behavioural reactivity) and parental feeding practices (feeding to regulate emotions) shape approach-avoidance tendencies at the neural level, and suggest that neural markers associated with approach-avoidance tendencies toward food may emerge much earlier than overt EE behaviours.

Our hypothesis that infants would display a stronger neural motivational approach to food stimuli in response to mild stress was partially supported by the data. We found that infants who displayed higher reactivity to the Lab-TAB “attractive toy behind the barrier” task showed stronger approach tendencies to food stimuli compared to non-food stimuli, as indicated by stronger left-than right hemispheric cortical activity. Yet, infants who reacted less to this task showed stronger approach tendencies to non-food items. This latter finding suggests that infants who display lower reactivity to a stressor may seek external non-food stimuli to help regulate their emotional state. Given that throughout infancy, a range of regulatory behaviours can be observed, such as attention diversion or exploration (Feldman et al., 2011), it is plausible that these infants already display early markers of adaptive regulation, pointing to an early capacity to recruit adaptive motivational processes for emotion regulation.

Conversely, infants with higher reactivity to a stressor may be more likely to associate a change in emotional state with food cues. This finding is in line with behavioural studies demonstrating that, for example, child negative affect is linked with their approach and avoidance behaviours towards foods (Liew et al., 2020, Steinsbekk et al., 2020, Zhou et al., 2019). EE behaviours may be observable in some children as young as 18 months (Chawner et al., 2025). However, as our study did not involve any direct observation of eating behaviour, we cannot conclude that infants with high reactivity demonstrated EE. Instead, our results point to a potential neural mechanism through which temperament may shape early food-related responses for some infants when exposed to an emotional perturbation.

We also found that feeding to regulate emotions predicted infants’ neural approach tendencies across conditions, thus partially supporting our initial hypothesis. This finding aligns with the well-established view that emotion regulation emerges through parent–infant co-regulation. Specifically, the link between emotional feeding and heightened approach tendencies is in line with previous eating behaviour research showing that parental use of food to soothe is associated with a child’s greater food responsiveness (Rodgers et al., 2014), emotional overeating (Hardman et al., 2016, Steinsbekk et al., 2018), and higher BMI (Jansen et al., 2019, Stifter et al., 2011). Given the correlational nature of the study, this association likely reflects an interplay between infant temperament and parental feeding practices, rather than a direct causal effect. For example, infants with certain temperamental profiles have parents who are more likely to use food to soothe (McMeekin et al., 2013, Stifter et al., 2011). Moreover, children with high negative affect are more likely to display food approach behaviours, which may trigger parental restrictive feeding practices, subsequently contributing to increased adiposity in the child (Liew et al., 2020). These patterns have often been interpreted in light of the differential susceptibility hypothesis (Belsky et al., 2007, Boyce and Ellis, 2005, Ellis et al., 2005), which suggests that children with increased physiological reactivity are more susceptible to both positive and negative environmental factors (although see Wass, 2018). Our results provide converging evidence that these associations may extend to the neural level and are already present in infancy.

Notably, emotional feeding predicted neural approach tendencies to both food and non-food stimuli, indicating that the association is not limited to food-related contexts. This pattern may reflect a broader reward sensitivity or a general motivational tendency toward external stimuli following emotional arousal. As distraction has been identified as an effective regulation strategy for young children (e.g. Feldman et al., 2011, Putnam et al., 2002, Segal and Moulson, 2024) and emotional feeding represents a form of external regulation, we can speculate that infants displaying neural approach tendencies after an emotional perturbation may be especially attuned to rewarding external stimuli. This could, in turn, reinforce parents’ use of emotional feeding as a soothing strategy, highlighting the reciprocal nature of early regulatory processes between parent and child.

Our hypothesis that food responsiveness would predict higher left frontal alpha asymmetry to foods than to non-foods was not supported by the data. Indeed, both infant satiety responsiveness and food responsiveness predicted reduced neural approach tendencies across conditions. These phenotypic appetitive tendencies, as assessed by parental reports, remain relatively stable across childhood (Ashcroft et al., 2008) and have been found to differently relate to prospective eating behaviour in children. Satiety responsiveness has been associated with lower child BMI, whereas food responsiveness has been linked to increased child weight gain (Olwi et al., 2023, Quah et al., 2015, van Jaarsveld et al., 2011, van Jaarsveld et al., 2014). Infants with high satiety responsiveness are thought to display the ability to regulate food intake in relation to satiety and this is considered a food-avoidance trait however, food responsiveness is considered a food-approach trait (Ashcroft et al., 2008). Therefore, while our result of an association between satiety responsiveness and reduced neural approach tendencies is in line with the literature, the association of food responsiveness with reduced neural approach tendencies is more difficult to unpack. We put forward some possible interpretations. First, as these associations were observed across conditions (food vs non-food images), it may be possible that the neural responses are shaped by broader cognitive or emotional factors rather than a direct reflection of appetitive tendencies. Second, we could also speculate that parental reports of appetitive traits do not fully align with neural responses in a controlled task at 12 months. As to our knowledge this is the first study to examine these associations in infants, further studies should specifically examine the interaction between food-approach and food-avoidance appetitive traits and FAA across infancy and childhood. Thirdly, these findings could be influenced by the task context. Since the task was conducted in the absence of hunger, food approach traits such as satiety responsiveness may have been reduced due to satiation. Therefore, linking points one and two, it is possible that in a state of hunger, rather than satiation, neural responses may more accurately reflect general appetitive traits.

The present study combined observational and neural measures to provide a comprehensive investigation through which to examine approach and avoidance tendencies associated with changes in physiological state and external stimuli. Although our measure of reactivity to the emotional perturbation could serve as a proxy of individual variations in interoceptive sensitivity, we did not directly measure infants’ sensitivity or accuracy to their internal sensations. Therefore, we cannot draw conclusions regarding the direct role of interoceptive processing in shaping approach and avoidance tendencies. Future studies could measure, for example, how gastric signals of hunger and satiety are represented in the infant brain and whether changes in emotional state may modulate this information.

In this study, we used standardised and controlled questionnaires and tasks, which bear the advantage of allowing for comparisons across different research studies. However, these measures may limit ecological validity. For example, it is possible that an infant’s response to the LAB-Tab task in our study may differ from their typical reaction to emotional stressors in real-world settings (see also Wass, 2018). Similarly, as our EEG task measured brain responses to images of food and non-food, these responses might have differed if the infants were presented with real foods and objects. Further studies should replicate our findings using more ecologically valid measures and stimuli.

To our knowledge, this is the first study to provide evidence that infants’ responses to change in physiological state are associated with approach-avoidance tendencies at the neural level, suggesting that these responses may influence how infants learn to interact with their environment. Although preliminary in nature, our results demonstrate that infants' behavioural reactivity and parental feeding practices may be closely linked to infants’ ability to form learned associations between internal states and external stimuli, a process that may lay the foundation for the development of appetite and emotional regulation skills. However, how parental influences may be associated with food approach tendencies remains unclear.

## CRediT authorship contribution statement

**Alejandra Sel:** Writing – review & editing, Formal analysis. **Arkadij Lobov:** Writing – review & editing, Formal analysis. **Sayaka Kidby:** Writing – review & editing, Writing – original draft, Methodology, Investigation, Data curation. **Liam R. Chawner:** Writing – review & editing, Writing – original draft, Methodology, Investigation, Formal analysis, Data curation. **Filippetti Maria Laura:** Writing – review & editing, Writing – original draft, Supervision, Project administration, Methodology, Investigation, Funding acquisition, Formal analysis, Data curation, Conceptualization.

## Declaration of Competing Interest

The authors declare that they have no known competing financial interests or personal relationships that could have appeared to influence the work reported in this paper.

## Data Availability

We have shared the link to the data
