# Supplementary material for: Neural basis of approach and avoidance responses to food in 12-month-old infants following emotional state changes
Source: Dev Cogn Neurosci. 2025 Dec 7;77:101656. doi: 10.1016/j.dcn.2025.101656 (PMC12757452; doi:10.1016/j.dcn.2025.101656)
Supplement: Supplementary material [file mmc1.docx]

**Supplementary Material –** Neural basis of approach and avoidance responses to food in 12-month-old infants following emotional state changes

1. **Information regarding the images used for the EGG food task.**

Images were sourced from Blechert’s database of food and non-food images for neuropsychological research. This database contains standardised stimuli (Blechert et al., 2014). The following image numbers were used, high energy density (HED) foods: 512, 536, 10, 131, 512, 536, 302, 556, 26, 27, 32, 310, 92, 515, 155, 193, 31, 4, 119, 15, 7, 135, 137, 25, 140, 101; low energy density (LED) foods: 192, 231, 200, 221, 341, 282, 234, 243, 206, 214, 215, 267, 197, 275, 288, 361, 305, 424, 270, 459, 307, 371, 560, 397, 543, 321. Image properties and statistics are displayed in the table below.

**Table 1S.** *Image properties and statistics of the images used for the EEG food task*

|  | HED food | LED food | Statistic |
| --- | --- | --- | --- |
| kCal total | 331.88 (150.58) | 72.19 (58.88) | t(25)= 9.39, *p* < 0.001 |
| kCal per 100 g | 498.10 (546.29) | 140.87 (138.64) | t(25)=3.22, *p* < 0.05 |
| Red | 0.47 (0.037) | 0.48 (0.09) | t(25)= -0.72, *p* = 0.48 |
| Green | 0.34 (0.022) | 0.34 (0.08) | t(25)= -0.32, *p* = 0.76 |
| Blue | 0.20 (0.040) | 0.18 (0.05) | t(25)= 2.03, *p* = 0.05 |
| Object size | 0.35 (0.10) | 0.310 (0.06) | t(25)=1.48, *p* = 0.15 |
| Intensity (normalised) | 92.32 (21.21) | 109.44 (37.55) | t(25)= -1.66, *p* = 0.11 |
| Contrast | 47.85 (14.83) | 44.86 (12.53) | t(25)= 0.99, *p* = 0.33 |
| Complexity (normalised) | 0.35 (0.10) | 0.32 (0.09) | t(25)= 1.30, *p* = 0.21 |

1. **Permutation analysis**

To examine the differences in FAA to observing food vs no-food items we adopted a non-parametric, cluster-based permutation approach. The analyses were restricted to two electrodes on the right (E124) and left (E24) hemisphere (Figure 1S), where the neural phenomena linked to the FAA are typically distributed. The FAA was computed by subtracting the signal recorded in the sensor over the left hemisphere (E24) from their corresponding sensor in the right hemisphere (E124).

Subject-wise activation time-courses were extracted with WTools and then passed to the statistical analysis procedure run in FieldTrip (https://www.fieldtriptoolbox.org/), the details of which are described by Maris and Oostenveld (Maris & Oostenveld, 2007; Oostenveld et al., 2011); Subject-wise activation time-courses were compared to identify statistically significant clusters in the time domain using a FieldTrip-based analysis across all time points within the selected time window. FieldTrip uses a nonparametric method (Oostenveld et al., 2011) to address the multiple comparison problem. T-values of adjacent temporal and frequency points whose p-values were less than 0.05 were clustered by adding their t-values, and this cumulative statistic is used for inferential statistics at the cluster level. This procedure, i.e. the calculation of t-values at each temporal point followed by clustering of adjacent t-values, was repeated 1000 times, with randomised swapping and resampling of the subject-wise time-frequency activity before each repetition. This Monte Carlo method results in a nonparametric estimate of the P-value representing the statistical significance of the identified cluster.

Specifically, we used the cluster-based permutation approach as implemented in Fieldtrip to test if the FFA changes varied when observing food vs non-food stimuli. Since this method allows the comparison of only two conditions, we first contrasted FAA differences between food and non-food stimuli and then performed a second analysis comparting LED vs HED food by means of within subject non-parametric cluster-based permutation analysis as described above. A non-parametric, cluster-based permutation approach is an efficient way of dealing with the multiple comparison problem that prevents biases in pre-selecting time-windows avoiding inflation of type I error rate. Given the lack of previous investigations examining the FAA in the context of a task, we took a conservative approach performing the statistical analyses across the entire time window ranging from 400ms to 850ms, where the natural phenomenon of the FAA emerges in the data (Figure 1S). For each comparison, subject-wise activations at selected electrode sites were extracted and passed to the analysis procedure. To avoid spurious findings, significant effects of 15 milliseconds or shorter were discarded from further analysis.


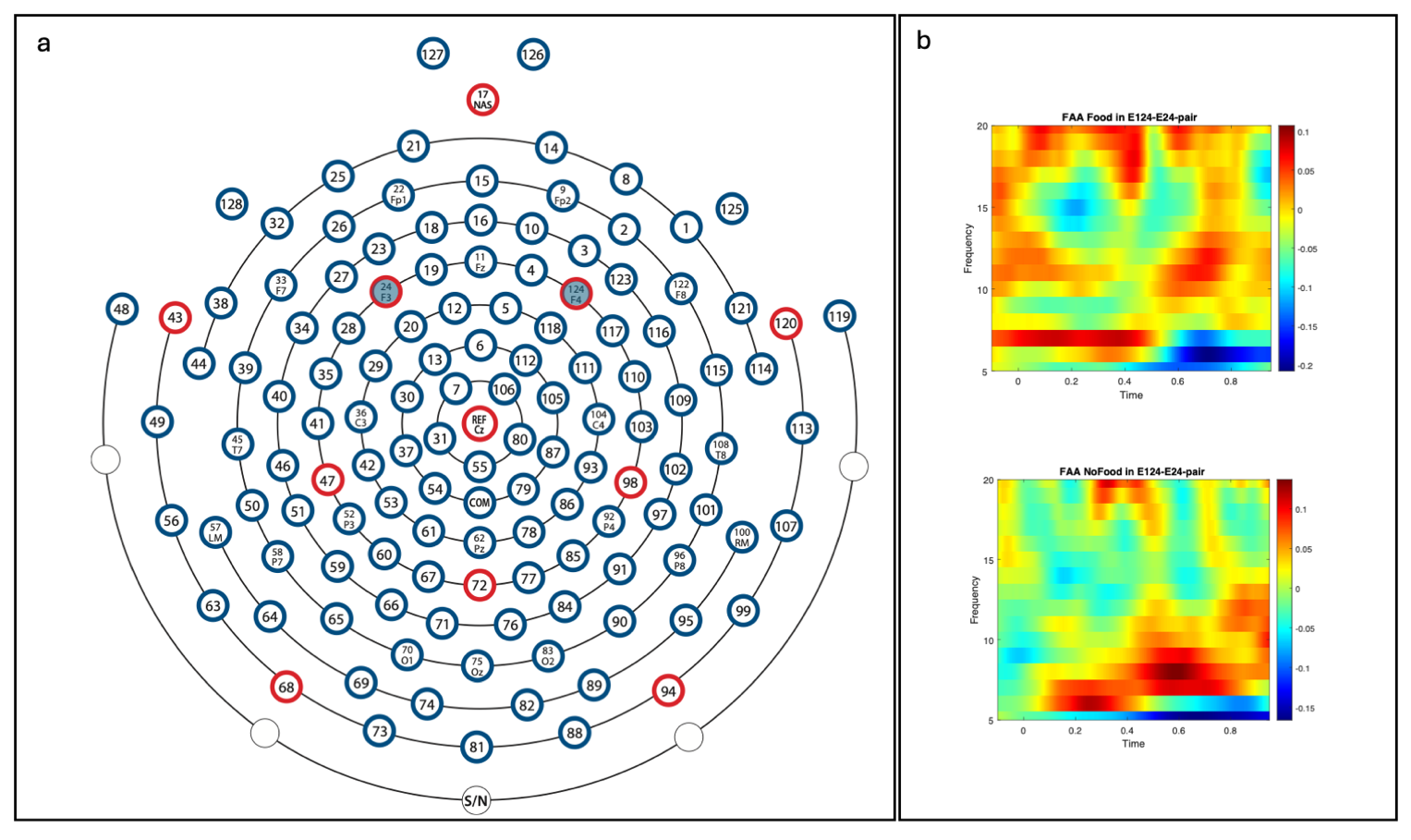
**Figure 1S.** EEG channel array and visualisation of FAA differences between food and non-food stimuli.

Note. (a) EEG 128-channel map with channels of interest in the left (E24) and right (E124) hemisphere. (b) Time-frequency power (5-20Hz) for the food condition (top-panel) and the non-food conditions (bottom panel) for the E24-E124 pair.

1. **Sensitivity analysis**

**Table 2S.** *Sensitivity analysis* *removing infants who ate ‘less than usual’ (N=21) in the lunchtime task. This is comparable to Table 2 in the main manuscript.*

|  | **Frontal Alpha Asymmetry (FAA)** | | | | |  |
| --- | --- | --- | --- | --- | --- | --- |
| *Predictors* | *Estimates* | *Std. Error* | *CI* | *t-value* | *p-value* | |
| (Intercept) | 0.87 | 0.18 | 0.54, 1.2 | 4.74 | **<0.001** | |
| Estimated lunch calorie  intake | 0.00 | 0.00 | 0, 0 | 0.01 | 0.996 | |
| Satiety responsiveness (CEBQ) | -0.22 | 0.04 | -0.29, -0.15 | -5.62 | **<0.001** | |
| Food responsiveness (CEBQ) | -0.19 | 0.03 | -0.25, -0.13 | -5.83 | **<0.001** | |
| Emotional overeating (CEBQ) | -0.19 | 0.08 | -0.33, -0.05 | -2.38 | **0.024** | |
| Feeding to regulate emotions (CFPQ) | 0.24 | 0.03 | 0.18, 0.3 | 7.63 | **<0.001** | |
| Use of food as a reward (CFPQ) | 0.09 | 0.05 | 0, 0.18 | 1.82 | 0.078 | |
| Behavioural reactivity (Lab-TAB) | -0.02 | 0.01 | -0.05, 0 | -1.73 | 0.095 | |
| Condition (Food) [Ref: Non-Food] | -0.36 | 0.13 | -0.59, -0.12 | -2.74 | **0.010** | |
| Behavioural reactivity (Lab-TAB) × Condition (Food) | 0.04 | 0.02 | 0.01, 0.08 | 2.12 | **0.043** | |
| Observations |  |  |  | N = 20 | |  |
| R^2^ / R^2^ adjusted |  |  |  | 0.53 / 0.39 | |  |
| F |  |  | F(9,30) = 3.73, **p = 0.003** | | |  |

1. **Exploratory analyses**

**Table 3S.** *Linear regression model showing the relationship between resting state FAA and child temperament.*

|  | **Resting state FAA** | | |
| --- | --- | --- | --- |
| *Predictors* | *Estimates* | *CI* | *p* |
| (Intercept) | -0.84 | -1.85 – 0.16 | 0.097 |
| Behavioural reactivity (Lab-TAB) | -0.03 | -0.06 – 0.01 | 0.136 |
| Surgency (IBQ) | 0.16 | -0.02 – 0.34 | 0.076 |
| Negative affect (IBQ) | -0.07 | -0.19 – 0.06 | 0.281 |
| Effort control (IBQ) | 0.08 | -0.09 – 0.24 | 0.352 |
| Observations | 45 | | |
| R^2^ / R^2^ adjusted | 0.131 / 0.044 | | |

**Table 4S.** *Linear regression model showing the relationship between resting state FAA, child eating behaviour and parental feeding practices.*

|  | **Resting state FAA** | | |
| --- | --- | --- | --- |
| *Predictors* | *Estimates* | *CI* | *p* |
| (Intercept) | -0.05 | -0.84 – 0.74 | 0.897 |
| Satiety responsiveness (CEBQ) | -0.15 | -0.36 – 0.05 | 0.134 |
| Food responsiveness (CEBQ) | -0.02 | -0.20 – 0.16 | 0.844 |
| Emotional overeating (CEBQ) | 0.13 | -0.14 – 0.40 | 0.332 |
| Feeding to regulate emotions (CFPQ) | 0.00 | -0.19 – 0.19 | 0.970 |
| Use of food as a reward (CFPQ) | 0.17 | -0.07 – 0.42 | 0.155 |
| Observations | 51 | | |
| R^2^ / R^2^ adjusted | 0.158 / 0.064 | | |

**References**

Blechert, J., Meule, A., Busch, N. A., & Ohla, K. (2014). Food-pics: an image database for experimental research on eating and appetite. *Frontiers in psychology*, *5*, 617.

Maris, E., & Oostenveld, R. (2007). Nonparametric statistical testing of EEG-and MEG-data. *Journal of neuroscience methods*, *164*(1), 177-190.

Oostenveld, R., Fries, P., Maris, E., & Schoffelen, J. M. (2011). FieldTrip: open source software for advanced analysis of MEG, EEG, and invasive electrophysiological data. *Computational intelligence and neuroscience*, *2011*(1), 156869.
